# A qualitative study of leaders’ experiences of handling challenges and changes induced by the COVID-19 pandemic in rural nursing homes and homecare services

**DOI:** 10.1186/s12913-024-10935-y

**Published:** 2024-04-09

**Authors:** Malin Knutsen Glette, Tone Kringeland, Lipika Samal, David W. Bates, Siri Wiig

**Affiliations:** 1 SHARE – Center for Resilience in Healthcare, Faculty of Health Sciences, University of Stavanger, Stavanger, Norway; 2 Department of Health and Caring Sciences, Western Norway University of Applied Sciences, Haugesund, Norway; 3 Division of General Internal Medicine and Primary Care, Department of Medicine, Brigham & Women’s Hospital, Harvard Medical School, Boston, MA USA; 4 Department of Health Policy and Management, Harvard T. H. Chan School of Public Health, Boston, MA USA

**Keywords:** COVID-19, Leader, Leadership, Nursing home, Home care, Resilience in healthcare, Patient safety, Quality of care

## Abstract

**Background:**

The COVID-19 pandemic had a major impact on healthcare services globally. In care settings such as small rural nursing homes and homes care services leaders were forced to confront, and adapt to, both new and ongoing challenges to protect their employees and patients and maintain their organization's operation. The aim of this study was to assess how healthcare leaders, working in rural primary healthcare services, led nursing homes and homecare services during the COVID-19 pandemic. Moreover, the study sought to explore how adaptations to changes and challenges induced by the pandemic were handled by leaders in rural nursing homes and homecare services.

**Methods:**

The study employed a qualitative explorative design with individual interviews. Nine leaders at different levels, working in small, rural nursing homes and homecare services in western Norway were included.

**Results:**

Three main themes emerged from the thematic analysis: “Navigating the role of a leader during the pandemic,” “The aftermath – management of COVID-19 in rural primary healthcare services”, and “The benefits and drawbacks of being small and rural during the pandemic.”

**Conclusions:**

Leaders in rural nursing homes and homecare services handled a multitude of immediate challenges and used a variety of adaptive strategies during the COVID-19 pandemic. While handling their own uncertainty and rapidly changing roles, they also coped with organizational challenges and adopted strategies to maintain good working conditions for their employees, as well as maintain sound healthcare management. The study results establish the intricate nature of resilient leadership, encompassing individual resilience, personality, governance, resource availability, and the capability to adjust to organizational and employee requirements, and how the rural context may affect these aspects.

## Background

In 2021, essential healthcare services in 90% of the world’s countries were disrupted by the COVID-19 pandemic [1]. Healthcare services were heavily stressed and had to address unexpected issues and sudden changes, whilst still providing high quality care over a prolonged period [2, 3]. Despite the intense focus on hospitals during this period, other parts of the healthcare system such as nursing homes and homecare services also faced extreme challenges. These included issues such as having to introduce and constantly adapt new infection control routines, as well as being given increased responsibility in caring for infected and seriously ill patients in facilities that were not built for such circumstances [4–7]. Mortality rates in nursing homes were especially high [8].

Resilience in healthcare is about a system’s ability to adapt to challenges and changes at different levels (e.g., organization, leaders, health personnel) to maintain high quality care [9, 10]. During the COVID-19 pandemic, leaders and the front line were forced to rapidly adjust to keep healthcare services afloat. It has been demonstrated in previous research that effective leadership is crucial in navigating crises and building resilience within health systems [11–13]. Furthermore, leaders play key roles in facilitating health personnel resilience, for example, through promoting a positive outlook on change and by developing health personnels’ competencies and strengths [12, 14, 15]. During the COVID-19 pandemic, this role became intensified [16–18], and leaders’ roles in promoting resilient healthcare services were central, for example safeguarding resources, providing emotional support and organizing systems to cope with extreme stresses [3, 19].

Smaller, rural nursing homes and home care services are geographically dispersed and typically remote from specialized healthcare services or other nursing home and homecare services. They also tend to have reduced access to personnel due to low population density, frequently leading to the need to make independent decisions, often in complex situations [20]. Overall, rural healthcare services face different challenges than their urban counterparts [21–23]. The COVID-19 pandemic intensified some of these issues and created new ones which needed to be managed [21, 24, 25].

The research base on COVID-19 has expanded extensively the past years [26], covering areas such as clinical risks and outcomes for healthcare workers [27] and patients [28], hospital admissions [29] and healthcare utilization during the pandemic [30]. Moreover, areas like healthcare leaders' [16, 17, 31] and healthcare professionals’ [2, 32] strategies to handle the pandemic challenges, and COVID related strategies’ effect on quality of care [33, 34]. And lastly, but not exhaustively, the COVID-19 pandemic in different healthcare settings such as hospitals [35], primary healthcare services and [36] mental healthcare services [37]. However, research on rural healthcare settings, particularly leaders in rural nursing homes and homecare services, have received less attention [38–40]. Despite the anticipated importance of primary healthcare services in future healthcare and the prevalence of rural healthcare options [41, 42]. Overall, there are still lessons to be learned from the COVID-19 pandemic, specifically identifying resilience promoting and inhibiting factors in different health care settings during crisis, how leaders deal with crisis management, and furthermore, to understand and draw lessons from challenges that were overcome during the pandemic[43, 44].

### Aim and research question

The aim of this study was to assess how healthcare leaders in rural primary healthcare services managed nursing homes and homecare services during the COVID-19 pandemic. Moreover, the study aimed to explore how adaptations to changes and challenges induced by the pandemic were handled by these leaders.

The research question guiding the study was: *How did primary healthcare leaders in rural areas experience their leadership during the COVID-19 pandemic, and how did they adapt to the rapid onset changes demanded by the COVID-19 outbreak?*

## Methods

### Design

The study employed a qualitative explorative design to study in-depth, how nursing home and homecare leaders in Norwegian rural primary healthcare services experienced and addressed the extreme challenges and needs for change induced by the COVID-19 pandemic [45, 46]. Four rural municipalities of different sizes were included in the study. Nursing home and homecare leaders at different organizational levels participated in individual interviews (See Table 1).

**Table 1:** Overview of participants and setting

**Municipality**	**Leaders**	**Setting**
Municipality A	1 Head of section 1 Ward leader	1 Nursing home
Municipality B	1 Municipal health and care manager 2 Unit leaders	1 combined nursing home and homecare service
Municipality C	2 Ward leaders 1 Head of district	1 nursing home 1 home care service
Municipality D	1 Municipal health and care manager	1 combined nursing home and home care
	Total: 9 leaders	

### Setting

**Fig. 1:**
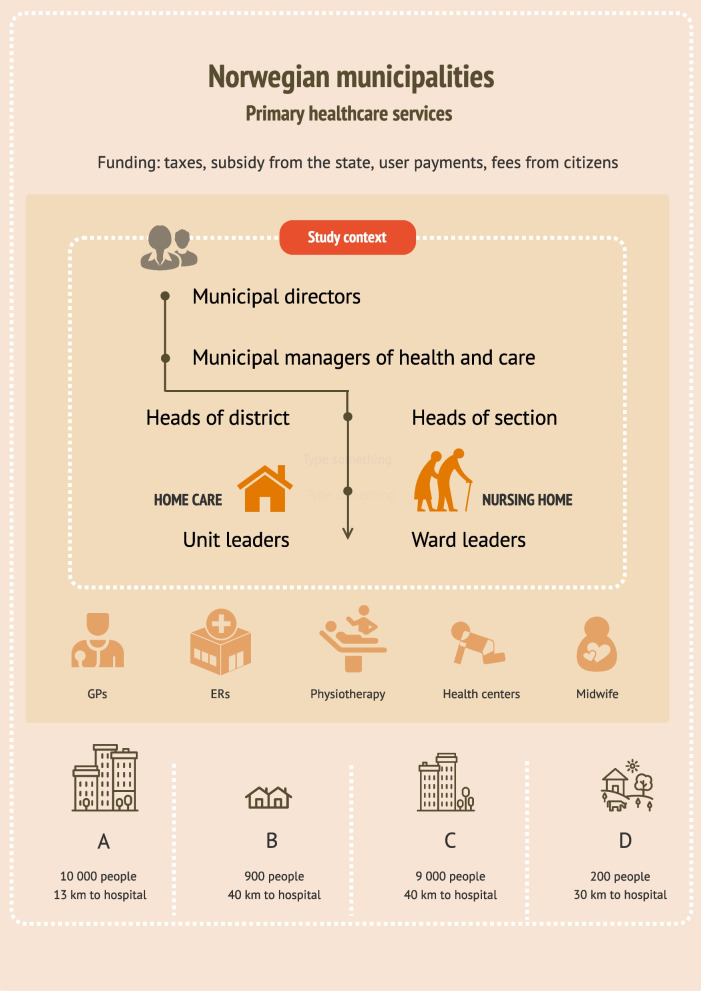
Brief overview of healthcare services provided by the Norwegian municipalities, comprising nursing homes and home care services, and the included municipalities

Norway is divided into 356 municipalities. These municipalities have the autonomy to administer and manage their primary healthcare services, subject to certain laws and regulations (e.g., Act on municipal health and care services [47], Act on patient and user rights [48] and Regulation on quality in nursing and care services for service provision [49]). All municipalities are obligated to offer specified healthcare services independent of their size and inhabitant number (Se Fig. 1 for a brief overview of healthcare services provided by the Norwegian municipalities, comprising nursing homes and home care services, and included municipalities).

### Recruitment and participants

Recruitment was anchored in the municipal management. The municipal manager of health and care in 11 municipalities across the Norwegian west coast were first contacted via email, then by telephone (se Fig. 1). Most managers who responded to our contact were positive, but many had to decline due to time constraints related to pandemic management. Four managers agreed to data collection in their municipality with the stipulation that the nursing home- and homecare leaders wanted to participate. All levels of leaders were eligible for inclusion due to the small size of the healthcare services. We contacted the leaders of nursing homes and home care services in the four municipalities, first by email, then by telephone. Nine leaders agreed to participate. One leader declined. All included leaders were female, registered nurses (RNs), and had long and broad experiences with working as RNs either in the healthcare service they now were leaders in, or in other healthcare settings. Some leaders stated that they had continued education or Master’s degrees, but more leader specific qualifications such as leader education, training or courses were not disclosed (Table 1. Overview of participants and setting).

### Data collection

Individual interviews were conducted from November 2021 to November 2022 by the first author (MKG). Leaders in one of the municipalities (municipality B) wished to do the interview in a group interview (three leaders), which we arranged. All but one interview was conducted at the leaders’ work premises (in their offices or in meeting rooms). One leader was interviewed via Zoom due to a temporary need for increased infection precautions. All interviews were guided by a predeveloped interview guide which was based on resilience in healthcare theory [50, 51] and contained subject such as: *Success factors and challenges with handling the COVID-19 pandemic; New solutions and how new knowledge and information was handled; and Lessons learned from the pandemic.*

### Data analysis

The interviews were audio recorded and transcribed. The analysis followed the steps in Braun and Clarkes thematic approach [52]. This involved reading through the transcripts multiple times to find meanings related to the overall research question. Text with meaning was inserted into a Word table which provided initial codes. After the coding process, which involved creating and continuously revising codes, there were 47 codes. The codes were then organized into categories and categories were sorted into initial main themes. Themes and categories were assessed to determine whether any of them should be merged, refined, split or eliminated [52] (see Table 2 for example of the analysis process). The author team reviewed and approved categories and themes to ensure that each theme illuminated its essence [52].

**Table 2:** Example of analysis process

Theme	**Categories**	***Codes***
**Navigating the role of a leader during the pandemic**	Personal challenges	*Fear of making mistakes*
		*Weighted down by responsibility and previous experiences*
		*Worrying about their employees*
		*Being “on call” for two years*
		*Experiencing a feeling of chaos*
		*Feeling worn out*
		*Taking care of each other within the leader group*
	Challenges related to the healthcare organization	*Dealing with a stressed economy*
		*Caring for next of kin*
		*Having to do many tasks in a limited amount of time*
		*A lack of preparedness*
		*Challenging access to PPE*
	Challenges in handling, distributing, and adapting to constant changes in information and routines	*Rapid changes in information and routines from the government*
		*Find and distribute right information at the right time*
		*Access to information and support*
		*Develop and distribute new routines*
	Enhanced responsibility for front-line employees	*Working to provide security among the employees*
		*Being visible*
		*Ensure understanding among the employees*
		*Handling displeased employees*
		*Being more strict than usual with rules and routines*
	Adapting to constant challenges and changes	*Planning for all possible scenarios*
		*Dealing with range of unfamiliar challenges*
		*Gaining control*
		*Maintain adequate staffing*
		*Introducing a range of measures to avoid spread of infection*
		*Introducing measures to avoid loneliness among patients*
		*Increased use of digital solutions*
		*Having to trust oneself*

## Results

We analyzed the interviews and identified three main themes and eight categories (Table 3). The results are presented according to identified main themes.

**Table 3:** Overview of identified main themes and categories

**Themes**	**Categories**
T1: **Navigating the role of a leader during the pandemic**	Personal challenges
	Challenges related to the healthcare organization
	Challenges in handling, distributing, and adapting to constant changes in information and routines
	Enhanced responsibility for front-line employees
	Adapting to constant challenges and changes
T2: **The aftermath—management of COVID-19 in rural primary healthcare services**	The positive experiences and outcomes
	The less positive experiences and outcomes
T3:** The benefits and drawbacks of being small and rural during a pandemic**	The uniqueness of being a small and rural municipality

### Navigating the role of a leader during the pandemic

Overall, the leaders seemed to have two primary focuses when they talked about how they had experienced the COVID-19 pandemic. These were their personal coping, and how they managed the organizational challenges arising throughout the pandemic period. Particularly in the beginning, they reported feelings of fear and insecurity. Leaders dreaded the consequences which could result from mistakes, such as providing wrong, or missing essential information.

“Having such a responsibility is a burden, and even though you’re not alone, you still feel like you’re the one responsible for the safety of the employees and the patients. Ensuring the safety of everyone was the priority, which is why it was critical to make sure that the protocols we were distributing were the correct ones…”

(L1 nursing home municipality C)

Additionally, several leaders stated that they were concerned about personnel who had contracted COVID-19 (some of whom had serious symptoms), and even felt responsible for their situation. Leaders of two of the municipalities reported feelings of frustration, and despair, and all leaders reported long working hours. Leaders expressed that they felt that they had been “on call” for the last two years, and described long working days, with limited consideration for evenings, nights, weekends, or vacations.

A range of organizational challenges was described (e.g., dealing with a stressed economy, experiencing task overload, working within an unprepared organization and the struggle to get a hold on enough personal protective equipment. One of the most prominent challenges in the data set, was the acquisition, interpretation, and distribution of information issued by the authorities. The leaders described that new information was issued frequently along with constantly changing routines. New routines where developed, distributed, and discarded nonstop in the attempt to “get the organization in line with the state authorities”.

“There was new information issued [from the Norwegian directorate of health] almost hourly… we had more than enough to, in a way, keep up with all these procedures that came, or all the new messages that came, and these [information and routines] had to be issued out to the employees and to the next of kin…”

(L1 nursing home municipality A)

Despite the difficulties related to information flow, or lack thereof, the leaders devised a range of solutions to make information more accessible to their staff (e.g., informational e-mails, developing short information sheets, making information binders, and meeting up physically to go through new routines with their employees). The data indicated that it was hard to gauge how much information to make available to their staff, who were eager for knowledge, yet still found it hard to process everything. On occasion, the leaders desired assistance or someone to assume authority, or as one leader articulated: “someone to push the red button” (L1 homecare municipality C), due to their struggles to keep up with information, regulations, and routines in the face of rapid changes.

Not surprisingly, leaders felt a heightened need to take the lead during the COVID-19 pandemic. This was a long-running crisis, and they had to be present, approachable and a source of support for their staff, while also striving to gain the employees’ understanding. For example, in one healthcare service the employees wanted more strict rules than necessary and had strong opinions on how things should be done in “in their healthcare service”, while the leader was stringent with sticking to national regulations which were less strict. Another aspect was handling disagreement with measures among employees. Often measures were not in line with the employees’ wishes, which created friction.

The pandemic highlighted the importance of leaders taking on the task of creating a secure working environment for their employees. The leaders noted considerable anxiety among the staff, particularly in facilities that had not experienced any COVID-19 cases. Leaders came to understand the importance of tending to all wards, regardless of whether they had been affected by the infection, even though it was perceived as taxing. Overall, the leaders worked actively to make the situation in wards with infection outbreaks as best as possible. A leader from a healthcare service which had a major COVID-19 outbreak stated:

“We constantly tried to create new procedures to make it as easy as possible for them [So] that they didn’t have to think about anything. That they [didn’t have to think about] bringing food to work, that they had to [remember] this or that. That they were provided with everything they needed…”

(L2 nursing home municipality C)

Another recurring topic in the dataset, was the constant challenges and changes the leaders had to overcome and adapt to during the COVID-19 pandemic. For example, there was a need to plan for all possible scenarios, particularly if they were to have a major infection outbreak among the staff (e.g., how to limit the infection outbreak, how to deal with staffing, how to arrange the wards in case of an outbreak). One healthcare service experienced such a scenario, which demanded a rapid response, when they had a major COVID-19 outbreak with over twenty infected employees almost overnight. The leaders were left with the impossible task of covering a range of shifts, and they were forced to adopt a strategy of reaching out to other healthcare services within their municipality (other wards, nursing homes, the home care services and psychiatric services) asking if they had any nurses “to spare.” Eventually, they managed to cover their staffing needs without using a temp agency.

The leaders of this nursing home also had to deal with numerous small, but important challenges such as how to deal with dirty laundry, what to do with food scraps, where to put decorations and knick-knacks, how to provide wardrobes and lunchrooms, and generally, how to handle an infection outbreak in facilities not designed for this purpose.

Leaders in all primary healthcare services implemented strategies to prevent infection or spread of infection. They introduced longer shifts, split up the personnel in teams, made cleaning routines for lunchrooms and on-call rooms, set up a temporary visiting room for next of kin, developed routines for patient visits, regularly debriefed personnel of infection routines, made temporary wardrobes, and removed unnecessary tasks from the work schedule. New digital tools were introduced, particularly for distributing instructional videos and information among employees, and to keep contact with other leaders.

Although many leaders described the situation as challenging, particularly in the beginning, many found themselves gaining increased control over the situation as time went by.

“Little by little, in some way, the routine of everyday life has become more settled… you can’t completely relax yet, but you can certainly feel a bit more organized, and more confident in your decisions, since we have been doing it for a while [ca 1 year].

(L1 nursing home municipality C)

### The aftermath—management of covid-19 in rural primary healthcare services

Despite organizational as well as personal challenges, leaders’ overall impression of the COVID-19 management was positive. The leaders firmly believed that the quality of healthcare services had been preserved, and all the physical healthcare needs of the patients had been properly cared for. According to leaders, there was not a rise in adverse events (e.g., falls, wounds) and patients and next of kin were positive in their feedback. The one main concern regarding quality of care was, however, the aspect of the patients’ sociopsychological state. Patients became isolated and lonely when they could not receive visitors or had to be isolated in their rooms or their homes during COVID. Nevertheless, the leaders expressed admiration for the healthcare personnel's work in addressing psychosocial needs to the best of their capacity. Overall, the leaders were proud of how the front-line healthcare personnel had handled the pandemic, and the extraordinary effort they put in to keeping the healthcare services running.

Several leaders stated that they now felt better prepared for “a next pandemic”, but they also had multiple suggestions for organizational improvements. These suggestions included: set up a visit coordinator, develop a better pandemic plan, be better prepared nationally, develop local PPE storage sites, introduce digital supervision for isolation rooms (for example RoomMate [53]), provide more psychological help for employees who struggled in the aftermath of an infection outbreak, have designated staff on standby for emergency situations, establish clear communication channels for obtaining information and, when constructing new nursing homes and healthcare facilities, consider infection control measures.

The leaders also discussed the knowledge they had acquired during this period. Many talked about learning how to use digital tools, but mostly they talked about the experience they had gained in handling crisis:

“I believe we are equipped in a whole different way now. There’s no doubt about that. Both employees and leaders and the healthcare service in general, I think… I have no doubt about that… so… there have been lessons learned, no doubt about it….”

(L1 nursing home municipality C)

Leaders also talked about what they experienced as success factors in handling the pandemic: Long shifts (11,5 h), with the same shift going 4 days in a row to avoid contacts between different shift, the use of Microsoft Teams and other communication tools to increased and ease intermunicipal cooperation, and the possibility to share experiences, making quick decisions and take action quickly, developing close cooperation with the municipality chief medical officer and the nursing home physician, the involvement of the occupational healthcare service (take the employees’ work situation seriously) and the conduct of “Risk, Vulnerability and Preparedness” analysis (a tool to identify possible threats in order to implement preventive measures and necessary emergency response). The leaders also talked about the advantages of getting input from employees (e.g., through close cooperation with the employee representatives).

### The benefits and drawbacks of being small and rural during a pandemic

Aspects of being a small healthcare service within a small municipality were highlighted by several of the leaders. For example, the leader of one the smaller healthcare service included in the study, addressed the challenge of acquiring enough competent staff. To be able to fulfill their requirements for competent staff, the municipality needed to buy healthcare services from neighboring municipalities. Another drawback was that employees who had competence or healthcare education often lacked experience in infection control and infection control routines, because they had rarely or never had infectious outbreaks of any kind. This made it particularly challenging to implement infection control measures. In one of the larger municipalities in this study, they had worked targeted for years to increase the competence in their municipality by focusing on full time positions to all and educating assistants to become Licensed practical nurses (LPN). They benefited from these measures during the pandemic.

Another aspect which was emphasized as essential to survive a pandemic in a small municipality, was intermunicipal cooperation. Leaders of all four healthcare services stated that they built increased cooperation with nearby municipalities during the pandemic. Leaders from the different municipalities met often, sometimes several times a week, and helped each other, shared routines, and methods, asked each other questions, coordinated covid-19 testing and developed intermunicipal corona wards, kept each other updated on infection status locally, and relied on each other’s strengths.

“We established a very good intermunicipal cooperation within the health and care services. We helped each other. Shared both routines and procedures, and actually had Teams meetings twice a week, where I could ask questions…and… we all had different strengths in the roles we held, not all of them [group members] were healthcare personnel either, and they had a lot of questions regarding the practical [handling of the pandemic]. At the same time, they [people who were not healthcare personnel] were good at developing routines and procedures, which they shared with the rest. In other words, the cooperation between the municipalities was very good, and for a small municipality, it was worth its weight in gold”.

(L1, nursing home/homecare Municipality D)

The same leader stated that they could not have managed the pandemic without support from other larger municipalities and advised closer cooperation following the pandemic as well. An advantage of being small was the ability to easily track and monitor the virus spread within the municipality. Moreover, it was easy to have close cooperation with the infectious disease physician, the municipal chief medical officer, and the nursing home physician, as one person often held several of these roles. Some leaders also had several roles themselves such as a combination of nursing home leader and homecare leader or a combination of nursing home leader and health and care manager (overseeing all health and care services in the municipality). This was perceived as both an advantage and a disadvantage. This was an advantage because they gained a full overview of the situation due to their multiple areas of responsibility, but a disadvantage because it was demanding for one person to handle everything alone, making the system vulnerable. Another challenging aspect was a lack of people to fill all the necessary roles. For example, in one municipality they did not have a public health officer (a physician in charge of the healthcare services in a municipality, and the municipal management’s medical adviser), and had to hire a private practicing physician, who was not resident in the municipality to take on this role.

The economy was also a continuous source of worry. Running a small healthcare service within a small municipality was stated as expensive because the municipalities were obligated to provide the same healthcare services as the larger municipalities, but with less income (e.g., tax payment per inhabitant). The pandemic led to new expenses such as overtime payment, and wage supplement for changed work hours. Leader had to continuously balance a sound use of resources, and responsible operation.

Table 4 provides and overview of the challenges leaders encountered, how they were handled, and leaders’ suggestions for further improvement.

**Table 4:** Overview of challenges, how they were handled and leaders’ suggestions for improvement

**Challenges**	**Measures/ Success factors**	**Suggestions**
	Creating a secure environment Taking care of employees’ basic needs Increased intermunicipal cooperation cooperation with the municipality chief medical officer and the nursing home physician Involvement of the occupational healthcare service	
**Extreme working hours**	Executed the work	
**task overload**	Executed the work	Get leader support constitute a visit coordinator
**unprepared organization**	Developing routines for patient visits Debriefing personnel of infection routines regularly Introducing longer shifts Splitting up personnel in teams Making cleaning routines Instructional videos “Risk, Vulnerability and Preparedness” analysis Made plans for: how to limit the infection outbreak, how to deal with staffing, how to arrange the wards in case of an outbreak	Be better prepared nationally Perform “Risk, Vulnerability and Preparedness” analysis A better national pandemic plan
**Lack of PPE**	Got a hold of PPE eventually	Have a local storage
**Information overload**	Information emails	Clearer information lines and an overview of where leaders can turn for information
	Developing short information sheets	
	Making information binders	
	physical appearance	
**The need to plan for all scenarios**	Close communication/collaboration with neighboring/larger municipalities	Continue communication/collaboration with neighboring municipalities/larger municipalities after the pandemic
**Understaffing under infection outbreaks**	Reach out to other healthcare services within their municipality (other wards, nursing homes, the home care services and psychiatric services Long shifts (11,5 h),	Have staff on call for emergency situations
**Handle infection outbreaks in buildings not designed for this**	Measures to deal with dirty laundry and other basics Remove all decorations and nick-knacks Split up or make, provisional wardrobes Split up or make, provisional lunchrooms Setting up a temporary visiting room for patients and next of kin,	Include thoughts of infection control when building nursing homes and healthcare institutions
**Lonely, iIsolated patients**	Applied for funding	Introduce digital supervision for isolation rooms

## Discussion

We assessed how leaders in rural primary healthcare services coped with unprecedented challenges during the COVID-19 pandemic. On one hand, they had to manage personal struggles such as insecurity, guilt, and excessive workload. At the same time, they had to confront major organizational issues such as financial instability, lack of resources, and information overload. Moreover, their roles changed, and the need to lead, make more decisions and be more supportive was heightened. While adapting to these changing roles, the leaders continuously introduced new measures to handle pandemic induced challenges including development of new routines, distilled and distributed information, reorganized staffing plans and rearranged wards. Although patients’ safety and quality of care was perceived as safeguarded throughout the COVID-19 pandemic period, leaders had several suggestions for improvements in case of future crises.

Previous research on primary healthcare services during COVID-19 support several of the findings identified here. Similar challenges requiring leaders to adapt their ways of working such as insufficient contingency plans and infection control, lack of staffing, changing guidelines and routines and challenges related to information flow were found [17, 31, 54–56]. Leader strategies to handle these challenges included reallocation of staff, providing support, provide training and distill and distribute information [16, 31, 55, 57]. Some findings in this study, particularly related to the rural context, has not been found elsewhere. We found that 1) the leaders’ and healthcare services’ increased their dependency on neighboring municipalities during the pandemic and 2) we identified both the advantages and drawbacks of leaders having to function in multiple roles during the pandemic. The heightened importance of cooperation within municipalities and healthcare services in rural areas as opposed to urban areas, has however, been highlighted both before and during the pandemic [17, 23].

The pandemic prompted organizations like the World Health Organization (WHO), International Council of Nurses (ICN), and Organization for Economic Co-operation and Development (OECD) to advocate for the advancement of more resilient healthcare services to be able to overcome current and future health system challenges [3, 58, 59]. To achieve the goal of resilient healthcare services, a multi-focal perspective incorporating both individual, teams and systems, is needed. This is because health system organization and leadership on all levels will impact how resilience can be built on team and individual level and thereby reinforce resilience in organizations [12, 51, 60–62].

### The multiple aspects of resilient leadership

Leadership style, leaders’ facilitation for flexibility and leaders’ management of resources, competence, and equipment, will affect the resilience of health personnel and thereby the organizational resilience [12, 15, 63]. However, resilient leadership is affected by multiple aspects. For one, leaders inherent individual resilience will influence how and if, they lead resiliently [64]. Individual resilience is a multifaceted concept consisting of the person’s determination, persistence, adaptability and recuperative capacity, and is impacted by their personal qualities, conduct and cultural outlook [12]. Similar to previous literature [65, 66], the current study found that leaders had to cope with personal challenges such as fear, guilt, adapting to changed roles and increased workload, while performing their everyday tasks. Literature have shown that leaders' responses to challenges can be influenced by their unique personality traits, ultimately shaping their resilience and leadership style [67, 68]. Personal qualities needed to “lead well” have also shown to vary between rural and urban healthcare services. For example, Doshi [69] found that being social, passionate and extrovert was more important in urban areas than in rural areas. This indicate that leaders’ personality traits affect resilience in healthcare, and that resilience promoting personality traits may vary across urban and rural areas. More research is needed to study these relationships.

Although measures to increase personal resilience can be effective (e.g., mindfulness, workshops/training, therapy) [70–73] it is not sufficient to base resilience building on these aspects alone [74]. There is a need to consider how leaders are influenced and supported by the system they are working within to become, and act more resiliently. This includes the support leaders have in their community (e.g., peer support, leader support and proper guidance), their access to resources and their freedom to make decisions [60, 75, 76]. In the current study, it appeared to be a connection between leaders’ coping and the amount of support they had from colleagues. In our interpretation, leaders who talked about their cooperation with others, also talked more positively of their COVID-19 experiences (e.g., how much they had learned or what they had accomplished, rather than how pressured and anxious they were). Similar results have previously been found. For example, leaders in Marshall and colleagues’ study [65] felt isolated and struggled to make sense of the situation (COVID-19 induced challenges), while leaders in Seljemo and colleagues’ study stated that support from other managers made it easier to cope with high workloads [31]. In smaller rural healthcare settings, obtaining support can be challenging due to the limited presence of leader colleagues in close proximity [77]. Additionally, Gray & Jones [78] suggests that resilient leaders are leaders who ask for help when needed. This indicates that leaders in more isolated areas may require more effort to form connections beyond their organization, and rural healthcare systems must afford greater attention to enabling peer networking (e.g., by providing time and resources).

Through recurrent intermunicipal, online meetings, leaders in the current study attained to initiate, and preserve contact with other leaders in other healthcare settings, much more than before the COVID-19 pandemic. This was particularly important for the smallest, most rural municipalities, where one leader held many roles, and was by one leader, stated as the main reason they were able to manage the COVID-19 pandemic in their primary healthcare service. The tendency to increase intermunicipal cooperation during this period, and the overall need for smaller, rural healthcare services to cooperate with others is found in other literature [23, 79]. However, mostly as collaboration within primary healthcare services, and not across organizations. Although recommended by leaders, it is not clear if this close contact has been maintained after the pandemic.

The governance leaders are working under will affect leaders’ possibility to lead resiliently. The governance allows for effective coordination of financing, resource generation, and service delivery activities, ensuring optimal system performance [80]. Yet, governing for resilience has proven to be a major challenge, because it requires systems to be both flexible and stable at the same time [76]. Flexibility presupposes systems’, health personnel’, and leaders’ ability to adapt to current conditions, and is essential for systems to cope with unpredictable, non-linear, and ever-changing social and environmental conditions. Conversely, stability must also be implemented to ensure that new policies are sustained and effective, and to stabilize expectations and promote coordination over time [76]. This means that leaders need flexibility to make their own decisions, as well as the stability that proper guidelines and direction provides [81]. In this study, some leaders reported experiencing chaos and loss of control when routines and guidelines lacked in the beginning of the pandemic. Similar results have been found among other healthcare leaders, as well as healthcare personnel [32, 66]. In contrast, the leaders’ need for flexibility to be able to adapt to the everchanging work environment brought on by the pandemic (examples in Table 3) was demonstrated in this, and other studies [16, 17]. It can, however, be argued that the balance between flexibility and stability is often skewed more towards flexibility in rural regions. Rural leaders must make unsupported decisions more often than urban leaders as they face higher demands and fewer available resources (such as competence, staff, and funding) [77]. This requires rural leaders to be more innovative and adaptable to current circumstances [23, 69, 77, 79]. That said, the availability of resources have shown to impact a system's flexibility, often by influencing the quality of its adaptations [2].

In low-resource healthcare settings across the globe, certain adaptations made to combat pandemic challenges ended up causing damage (e.g., reuse or misuse of PPE, overexploitation of healthcare personnel and the use of unconventional treatment methods) [2, 82]. In high resource healthcare services, as included in this study, adaptations were often described as beneficial, and potential long-lasting solutions (Table 3) [16, 17, 31]. Although not comparable to low resource healthcare services, variation in resource availability and economy between the included healthcare services was also expressed in this study. Norwegian municipalities’ income is closely tied to their tax revenue and population size [83], and regardless of income, the municipalities are required to provide specific healthcare services to their inhabitants. Thus, the financial foundation of smaller more rural municipalities is not as strong as that of larger municipalities. These inequalities were expressed as notable by both leaders and by healthcare personnel in a preceding study exploring the same primary rural healthcare services as included here [32]. Since resilience in healthcare is also highly dependent on the competence and experience of employees and leaders, the combination of resource and financial deficiencies, more often experienced in rural healthcare services than in urban healthcare services, may pose particular challenges in resilience building in rural areas [23, 84]. This is worth exploring further, along with the rural healthcare services’ particular need to be flexible versus the potential difficulty they may have in making beneficial adaptations because of a weaker financial foundation.

### Resilience and leadership style

Providing support to employees was an important leader task during the pandemic [55, 66] and have further, been found to be particularly vital in rural areas, where employees have a smaller network of colleagues to turn to [84]. Other vital leadership tasks, recognizable from crisis leadership literature and also found in this study, were the importance of organizing, directing and implementing actions, forging cooperation, enabling work- arounds or adaptation, direct and guide and the importance of communication and dissemination of information [85, 86]. Although charismatic leadership^1^ has been found to be most valuable during crisis [87], there is an ongoing discussion of what leadership style is best suited to promote resilience in healthcare [11, 14, 66, 88]. For example, both transformational and transactional leadership^1^ [89] have been stated as resilience promoting leadership styles [15]. However, as found in other literature [66, 88], the results of this study indicated that leaders oscillated between different styles during the COVID-19 pandemic period. For example, in the beginning of the pandemic when uncertainty characterized the healthcare system, leaders became stricter with rules and regulations, demonstrating an authoritative leadership style^1^. Further, stepping in, lecturing about infection control procedures and use of PPE, indicated a coaching leadership^1^ style and lastly, when the leaders went against employees wishes to ensure safe maintenance of operation, it showed similarities to a transformational leadership style^1^ [90]. Interestingly, leaders did not speak directly about how their leadership styles changed, and seemed unaware of their leadership style adaptation. Similarly, in Sihvola et al. [66] leaders found it surprising how novel conditions could influence their leadership style.

On one side, these results, suggest that an adaptive leadership style can be necessary during crisis. On the other side, this and other studies [31, 54] indicate that leaders need more knowledge on crisis leadership, for example, to be made aware of the potential need to oscillate between different leadership styles during a crisis, and the possible subsequent challenges. For example, a study conducted by Boyle og Mervin [91] found that being a “nurse leader” (all leaders in this study were nurses), showed challenging because the leaders were judged as a peer rather than a leader. This can cause challenges, particularly when stepping into an authoritative leadership style. Such conflicts were not reported in this study, however, these are all aspects which should be given more attention when investigating resilience in healthcare and leadership styles [88]. Furthermore, it is crucial to acquire further understanding on the distinctions between leading in rural and urban areas, and how various leadership approaches may be impacted by managing tight-knit employee teams, which is often the case in small rural nursing home and homecare services. And finally, there is a need to provide a deeper understanding of the factors that promote or impede resilience in rural primary healthcare services, and the influence of the contextual aspects on resilience in healthcare.

### Limitations

This study has limitations which need to be addressed. A larger number of included primary healthcare leaders over a wider geographical area and across boarders would have provided a broader view of leader experiences during the COVID-19 pandemic. However, it was very difficult to get leaders to take time to reflect during this crisis. This study does provide insight into a variety of different municipalities of different sizes, organization and locations in the Norwegian context, providing a variety of rural primary healthcare leaders experiences during the pandemic. Interviews were conducted in different ways (focus group, digital and individually) this could have influenced leaders description of their experiences. Furthermore, interviews were held at different points throughout the pandemic phases, leading to a mix of leaders with both current and reflective experiences of navigating the pandemic. This should be taken into consideration when reading the results.

## Conclusion

By exploring nursing home and home care leaders’ experiences with the COVID-19 pandemic in rural areas, we found that the leaders met a range of rapid onset challenges of different nature, many of which demanded fast decisions and solutions. Leaders handled these challenges and changes in a variety of ways in their different contexts. In addition to health system challenges, leaders also had to cope with rapidly changing roles, while managing their own and employees’ insecurities. This study’s results demonstrate the intricate nature of resilient leadership, encompassing individual resilience, personality, governance, resource availability, and the capability to adjust to organizational and employee requirements. In addition, there may be differences between how resilience in healthcare is built and progresses in rural healthcare services versus urban contexts. Further research to understand the interplay between these aspects is needed, and it is critical to consider context.

## Data Availability

Data are available from the corresponding author upon reasonable request.

## Abbreviations

ICN: International Council of Nurses
LPN: Licensed practical nurse
OECD: Organization for Economic Co-operation and Development
PPE: Personal protective equipment
RN: Registered Nurse
WHO: The World Health Organization

## Footnotes

1 *Charismatic leadership*: influence and persuasion of others to help the fulfill their mandate, also in face of adversity; *Transformational leadership:* pushing to work and think in new ways; *Authoritative leadership*: the leader in control, low autonomy; *Coaching leadership*: the leader support employee’s skill advancement; *Transactional leadership*: exchange of rewards for fulfilling expectations.
